# The Estimation of Prevalence, Incidence, and Residual Risk of Transfusion-Transmitted Human Hepatitis B Infection from Blood Donated at the Anhui Blood Center, China, from 2009 to 2011

**DOI:** 10.1371/journal.pone.0073472

**Published:** 2013-09-13

**Authors:** Wuping Li, Zhan Gao, Chunhui Yang, Jiahu Li, Ling Li, Rong Lv, Zhong Liu

**Affiliations:** 1 MOH Key Laboratory of Systems Biology of Pathogens, Institute of Pathogen Biology, Chinese Academy of Medical Sciences & Peking Union Medical College, Beijing, China; 2 Institute of Blood Transfusion, Chinese Academy of Medical Sciences and Peking Union Medical College, Beijing, China; 3 Anhui Blood Center, Anhui, China; Centers for Disease Control and Prevention, United States of America

## Abstract

**Background:**

The high prevalence of hepatitis B virus (HBV) among the Chinese population poses a threat to blood safety; however, few studies have examined epidemiological data regarding HBV infection of Chinese blood donors. The present study investigated the demographic characteristics of blood donors at the Anhui blood center in China, the prevalence, incidence, and residual risk (RR) associated with hepatitis B surface antigen (HBsAg) expression in terms of transfusion transmitted HBV (TTHBV) infections.

**Methods:**

The demographic characteristics and HBV status of people who donated blood at the Anhui blood center between 2009 and 2011 were retrospectively analyzed. The incidence of HBV was estimated through HBsAg yield approach. The window period model was then used to estimate the RR of TTHBV infection.

**Results:**

The typical donor at the Anhui blood center was a first-time volunteer, aged less than 25 years, unmarried, of Han ethnicity, and with an education below high school level. The prevalence of HBV infection among repeat donors, first-time donors, and all donors was 28.9, 127.2 and 82.1 per 100,000, respectively. The incidence estimate was 333.9 per 10^5^ person-years. Using an infectious window period of 59 days, the RR for HBV was estimated to be 1 in 1853 between 2009 and 2011.

**Conclusions:**

The incidence and RR of HBV in Chinese blood donors are much higher than those of donors in developed countries. This is because sensitive ELISAs and nucleic acid tests are not available in China. Further work is needed to improve both the safety and availability of blood products in China.

## Introduction

Over the past 30 years, the risk of transfusion transmiitted HBV (TTHBV) has been markedly reduced by the development of increasingly sensitive hepatitis B surface antigen (HBsAg) tests, the adoption of antibody against hepatitis B core antigen (anti-HBc) screening by some countries, the use of nucleic acid amplification tests (NAT), and improved screening of volunteer donors [1,2,3]. However, the risk for TTHBV remains higher than that for hepatitis C virus (HCV) and human immunodeficiency virus-1 (HIV-1), despite the introduction of NAT by a number of countries [4,5].

HBV screening practices vary from country to country both in terms of the specific markers examined and the types of test used, and are often dependent on the level of healthcare resources. Anti-HBc screening could be implemented as an additional safety feature in countries with a low prevalence of HBV; however, such measures would lead to the rejection of many otherwise acceptable donors in countries with a moderate-to-high prevalence. TTHBV infection via blood donated by HBsAg-negative donors is a risk when the blood is collected during the window period (WP) or at the late stage of infection. This is a particular problem in countries with a high prevalence of HBV [6,7]that do not routinely use NAT; NAT can reduce the WP and identify occult HBV infections (OBI) [4,6]. It is estimated that more than 60% of the Chinese population had been exposed to HBV before the implementation of an infant HBV vaccination program in 1992, with an estimated 9.8% of the population being HBsAg-positive. Fortunately, the HBsAg-positive rate in the general population fell to 7.2% by 2006 [8,9], suggesting that vaccinating against HBV plays an important role in reducing HBV transmission. However, data pertaining to the prevalence, incidence, and residual risk (RR) of HBV in Chinese blood donors are limited. A previous study used two different ELISA kits to examine HBsAg levels in donated whole blood collected at four different centers in China between January, 2000, and December, 2010 [10]. The results indicated that the overall prevalence of HBsAg was 0.86%: eight times lower than that in the general population. However, this may be an underestimate, as pre-donation testing for HBsAg was both rapid and insensitive. To date, very few studies have examined the incidence of HBV in Chinese blood donors and the RR for HBV after blood transfusions. In China, blood banks screen donated blood using EIA tests; therefore, the risk of HBV transmission via blood obtained from HBV-positive donors during the WP remains relatively high. One study estimated the rate of OBI after blood transfusions to be 0.13% (5 of 2972 people) [11].

It is difficult to estimate the incidence and RR of HBV in China. This is due to the transient nature of HBsAg expression, a lack of routine testing for anti-HBc antibodies, and the HBsAg neutralization assays. Therefore, the present study reviewed all routine screening data obtained at the Anhui blood center from January, 2009 to December, 2011. We then performed additional HBsAg neutralization assays and anti-HBc tests on all samples that were positive for HBsAg and estimated the incidence of HBV infection using the HBV yield approach described by Zou et al. This approach uses the HBsAg and anti-HBc test results to estimate the incidence of HBV infection [12]. The RR of HBV infection via blood supplied by the Anhui blood center was then evaluated.

Thus, the aims of the present study were to examine the prevalence and incidence of HBV infection among blood donors at the Anhui blood center based on the results of anti-HBc and HBsAg neutralization assays, to estimate the RR of TTIs in this region, and to develop a reliable index on which to base future testing policy.

## Materials and Methods

### Ethics statement

The study was approved by the Ethics Committee of the Institute of Pathogen Biology, Chinese Academy of Medical Sciences & Peking Union Medical College. Written informed consent was obtained from each study participant before the interview, sample collection and testing.

### History questionnaire and rapid pre-donation screening

To identify donors during the potential WP of infection and reduce the costs associated with screening, all Chinese blood donors are required to pass a routine pre-donation screening process according to standards established by the Chinese Ministry of Health (MOH), which comprises a medical history questionnaire, pre-donation rapid screening, and a brief physical examination. The medical history questionnaire comprises the screening fields mandated by the Chinese Ministry of Health [13]. Briefly, all donors must declare any history of sexually transmitted diseases or hepatitis, illegal drug use, or sex with multiple partners. Male donors must also state whether they have ever had sexual intercourse with another man. Positive responses to these questions result in permanent deferral. Before collecting blood, all donors underwent rapid testing at the collection sites for hepatitis B surface antigen (HBsAg; colloidal gold strip method) and rapid tests for alanine aminotransferase (ALT). The rapid testing procedures also ensure that donors with increased ALT levels or a positive HBsAg result are temporarily deferred. Donors must also pass a physical examination, during which body temperature, weight, blood pressure, and hemoglobin levels are measured. The information of those rapid testing kits is demonstrated in Table 1.

**Table 1:** Test kits used in this study.

**Kit Name**	**Company**	**Sensitivity/specificity (%**)	**The low detection limit**
Diagnostic Kit for HBV Surface Antigen(ELISA)	Beijing WANTAI Biological Pharmacy Enterprise Co., Ltd.	99.5/98.5	0.03ng/ml
	bio-Rad Clinical Diagnostics	100/97.4	ND
HBsAg Confirmatory, Antibody to Hepatitis B Surface Antigen (Human)	Livzon Pharmaceutical Group Inc.	ND	ND
Diagnostic Kit for Antibody to Hepatitis Core Antigen(ELISA)	Beijing WANTAI Biological Pharmacy Enterprise Co., Ltd.	99.4/99.1	
hepatitis B surface antigen rapid test kit	Zhejiang Aikang Bio-technoly Co., Ltd	95.9%/99.8	1ng
ALT rapid test kit	Shanghai Rongsheng Biological Pharmaceutical Co., Ltd	ND	ND

ND: no data.

### Routine transfusion-transmitted infection test

Anhui blood center uses the standard blood donor recruitment and testing procedures approved by Ministry of Health of the People’s Republic of China [14]. Briefly, a unit of blood unit is collected and a sample is subjected to two rounds of EIA testing using two different reagents to measure ALT and HBsAg levels, and anti-HIV-1/2, anti-HCV, and anti-syphilis antibody. All test kits are approved and licensed by the Chinese State Food and Drug Administration. The reagents used for donor screening are listed in the Table 1, and the tests were prerformed according to the manufacturer’s instructions. The low limit of the detection of HBsAg kit (Wantai) is 0.030ng/ml. Screening reactivity is defined by a reactive result in one or both rounds of screening tests and if one round is negative, the sample is tested again using the test that gave a negative result. Between 2009 and 2010, the signal-to-cutoff ratio for the HBsAg test was set at 1.0; however, the value was changed to 0.5 from January, 2011.

### Confirmatory and supplementary testing

Seropositivity for HBsAg was assigned according to the results of confirmatory tests (neutralization assays performed by the Anhui blood center) as specified in the Table 1. Seropositivity was defined consistently throughout the study and was based upon recommendations made by the manufacturer of the HBsAg test kit. Samples confirmed as HBsAg-positive by the neutralization assay were then tested for anti-HBc antibodies as indicated in the Table 1.

### Statistical analysis

First-time donors were defined as donors who had not previously attended Anhui blood center. Repeat donors were defined as donors who had made previous donations according to the database. Details regarding the testing of previous donations made by positive donors were obtained so that they could be accurately classified as first-time donors or repeat donors.

HBsAg-positive donors were defined as those who donated a unit of blood that was subsequently confirmed as HBsAg-positive in the neutralization assay. To identify the prevalence of HBsAg, the number of donors was determined for each year (from 2009 to 2011). The numerator represented the number of donations (first-time and/or repeat) that were confirmed as positive, and the denominator represented the total number of donations (first-time and/or repeat) for which a test result (positive or negative) was available.

The incidence of HBV was determined using the HBsAg yield approach as previously described [12,15]. Early during an acute HBV infection, HBsAg becomes detectable before anti-HBc. Although HBsAg may be transient, anti-HBc persists after becoming detectable. Therefore, donations confirmed as HBsAg-positive but anti-HBc-nonreactive represent incident cases that had not yet seroconverted to anti-HBc antibodies; such cases were regarded as HBsAg yield cases. The number of HBsAg yield cases divided by the total number of donations is equal to the HBsAg yield rate which, when divided by the length of time a patient has been HBsAg-positive prior to anti-HBc seroconversion (termed the HBsAg yield window; calculated as 44 days), gives an incidence estimate for HBV infection among blood donors.

The RR attributable to WP donations was calculated using the following equation [5]:

RR=WP×incidencerate

Because Anhui blood center uses a low-sensitivity EIA kit for HBsAg testing, a WP of 59 days was used to estimate the length of time from the start of theoretical infectivity [5,16].

Data processing and analysis were performed using SAS computer software (SAS, SAS Institute, Cary, NC) [17]. An approximate 95% confidence interval (95% CI) was obtained using Monte Carlo simulation and Crystal Ball computer software (Crystal Ball, Decisioneering, Denver, CO) [18]. Statistical analyses were conducted using SPSS 11.5 statistics software. The Chi-square test was applied to assess the association between the categorical variants. A P-value of < 0.05 was used as the cut-off level for significance.

## Results

### Demographic characteristics of blood donors

From January 1, 2009 to December 31, 2011, 268,414 whole blood donations were collected at Anhui blood center. The demographic characteristics of the donors are presented in Table 2. Approximately 70% of donations were made by first-time donors and 30% by repeat donors. The proportions of male and female donors were 60% and 40%, respectively. Approximately 62.8% of donors were 25 years old or younger. The majority of donors were of Han ethnicity (99.5%) and 70.7% were single. In all, 41.7% of donors had an education below high school level, 66% weighed less than 65 kg, 36.1% were students, and 39.7% were in employment. Approximately 53.6% of donations were collected at mobile collection sites. There were no significant differences in these donor characteristics over the 3-year study period. Compared with those in the United States, Chinese donors are more likely to be young, first-time donors with a low level of education.

**Table 2:** Demographic characteristics associated with all donors in Anhui from 2009 to 2011.

	**2009 (%)**	**2010 (%)**	**2011 (%)**	**Total (%)**
**Number of donors**	**N=79.619**	**N=93,797**	**N=94,998**	**N=268,414**
**Donor status**				
**First-time**	**67.9**	**69.6**	**69.5**	**69.4**
**Repeat**	**32.1**	**30.4**	**30.5**	**30.6**
**Sex**				
**Male**	**54.7**	**60.3**	**61.8**	**59.2**
**Female**	**45.3**	**39.7**	**39.2**	**40.8**
**Age group(years**)				
**18-<25**	**70.2**	**64.5**	**55.0**	**62.8**
**26-<35**	**18.5**	**21.1**	**25.4**	**21.9**
**36-<45**	**8.6**	**10.8**	**14.6**	**11.5**
**46-<55**	**2.7**	**3.6**	**4.9**	**3.8**
**>55**			**0.1**	**0.1**
**Marriage status**				
**Single**	**78.8**	**72.0**	**62.7**	**70.7**
**Married**	**19.1**	**24.3**	**35.1**	**26.6**
**Miss**	**2.2**	**3.7**	**2.2**	**2.7**
**Ethnicity**				
**Han**	**99.4**	**99.5**	**99.5**	**99.5**
**Non-Han**	**0.6**	**0.4**	**0.5**	**0.5**
**Weight(kg**)				
**45-<55**	**33.5**	**28.9**	**26.7**	**29.5**
**56-<65**	**37.0**	**37.2**	**35.4**	**36.5**
**66-<75**	**19.5**	**22.1**	**24.0**	**22.0**
**76-<85**	**7.4**	**8.7**	**10.3**	**8.9**
**86-<95**	**2.1**	**2.5**	**2.9**	**2.5**
**Occupation**				
**Student**	**42.8**	**36.4**	**30.0**	**36.1**
**Working**	**29.4**	**33.9**	**54.3**	**39.7**
**Missing**	**27.8**	**29.7**	**15.7**	**24.2**
**Education**				
**Master**	**1.0**	**1.3**	**1.5**	**1.3**
**Bachelor’ degree**	**23.4**	**24.1**	**22.1**	**23.2**
**High school and associate degree**	**35.8**	**33.5**	**32.4**	**33.8**
**Below high school**	**39.8**	**41.1**	**44.0**	**41.7**
**Donation site**				
**Collection vehicle**	**54.6**	**52.9**	**53.5**	**53.6**
**Fixed site**	**45.4**	**47.1**	**46.5**	**46.4**

Data are reported as percentages

### Serologic prevalence of HBV as confirmed by the neutralization assay

The serologic prevalence of confirmed HBV infection of donations made by first-time and repeat donors is presented in Table 3. The HBV prevalence among first-time donors was 4.4-fold higher than that among repeat donors over the study period. However, during 2009 and 2010, the prevalence among first-time donors was approximately 6-fold higher than that among repeat donors, whereas the difference between the two groups in 2011 was 3-fold. This may be due to the fact that the signal-to-cutoff ratio for HBsAg was changed from 1.0 to 0.5 in 2011; HBV vaccination program implemented in 1992 and the first batch of vaccinated population reach the donation age at 2011 may also contributed to this change.

**Table 3:** HBsAg prevalence among blood donations in Anhui from 2009 to 2011.

	**2009**	**2010**	**2011**	**Total**
	**Prevalence (per10^5^py**)** (95%CI**)	**Prevalence (per10^5^py**)** (95%CI**)	**Prevalence (per10^5^py**)** (95%CI**)	**Prevalence (per10^5^py**)** (95%CI**)
**Repeat**	19.9 (7.6-32.2)	25.8 (12.8-38.9)	38.5 (21.2-55.8)	28.9 (19.7-36.1)
**First-time**	128.7 (98.8-158.6)	147.0 (117.4-176.5)	108.9 (85.2-132.5)	127.2 (111.3-143.0)
**Total**	76.9 (60.1-93.6)	89.6 (72.9-106.4)	80.8 (64.9-96.6)	82.7 (73.2-92.2)

The demographic characteristics of the HBsAg-positive donors are presented in Table 4. The overall rate of serologic-positivity varied according to donor status, gender, age, marital status, body weight, and educational level. HBV prevalence was 2-fold higher in first-time donors than in repeat donors. The prevalence was higher among males than females, although the difference was not statistically significant. Donors aged less than 25 years had a significantly lower rate of HBV-positivity. Positivity increased with age (0.09% in those aged 18–25 years and 0.24% in those aged 46–55 years, P<0.05). Marital status was also associated with positivity: the prevalence in married donors was twice that in single donors. A significantly higher positive rate was also observed in donors weighing 86–95 kg (P<0.05). There were no statistically significant differences in positivity in terms of occupation. The positive rate among blood donors with an educational level below high school was significantly higher than that among donors with a higher educational level (P<0.05). The positive rate among Han Chinese was slightly lower than that among minority Chinese.

**Table 4:** Demographic characteristics of HBsAg positive donors in Anhui from 2009 to 2011.

	Total*	
	**(number of positive donation/total donation**)	
**Donor type**		
**First-time**	247/193214 （0.13)	
**Repeat**	44/75200 （0.06)	
**Sex**		
**Male**	183/158825 （0.12)	
**Female**	108/109589 （0.10)	
**Age group(years**)		
**18-<25**	154/168596 （0.09)	
**26-<35**	62/58657 （0.11)	
**36-<45**	51/30828 （0.17)	
**46-<55**	24/10170 （0.24)	
**Marriage status**		
**Single**	164/189793 （0.09)	
**Married**	127/71322 （0.18)	
**Ethnicity**		
**Han**	289/267072 （0.11)	
**Non-Han**	2/1342 （0.14)	
**Weight(kg**)		
**45-<55**	76/79182 （0.10)	
**56-<65**	107/97971 （0.11)	
**66-<75**	59/59051 （0.10)	
**76-<85**	31/23888 （0.13)	
**86-<95**	12/6710 （0.18)	
**Occupation**		
**Student**	87/96772 （0.09)	
**Working**	118/106673 （0.11)	
**Missing**	86/64969 （0.13)	
**Education**		
**Master**	3/3475 （0.09)	
**Bachelor’ degree**	58/62264 （0.08)	
**High school and associate degree**	85/90697 （0.09)	
**Below high school**	145/111978 （0.1)	

* Data are reported as percentages

### Estimated incidence of HBV infection using the HBsAg yield approach

To determine the incidence of HBV incidence according to the HBsAg yield data, we used an HBsAg yield window of 44 days as described in a previous study [12]. The ratio for the verified HBsAg yield rate between donations from first-time donors and those from repeat donors was 6.82. Based on the verified HBsAg yield, the estimated incidence among repeat donors, first-time donors, and all donors was 79.3 per 10^5^ py (95% CI = 39.2–119.4), 540.6 per 10^5^ py (95% CI = 446.3–635.0), and 333.9 per 10^5^ py (95% CI = 278.8–389.0), respectively (Table 5).

**Table 5:** Incidence estimates based on verified HBsAg yield donations to Anhui from 2009 to 2011.

**Donor type**	**Total number Donations (1**)	**% of total donations**	**HBsAg yield (2**)	**Yield rate (/100,00**)** (3=2/1**)	**Ratio of Yield rate** *	**Yield window** ^#^ **(4**)	**Incidence (per10^5^py**)	
							**Estimate(3/4**)	**95%CI**
**Repeat**	157,690	44.8	15	9.5	1.00	0.12	79.3	39.2-119.4
**First-time**	194,214	55.2	126	64.9	6.82	0.12	540.6	446.3-635.0
**Total**	351,904	100	141	40.1		0.12	333.9	278.8-389.0

* Yield rate for first-time or total donor divided by yield rate for repeat donors

# Yield window was 44 days divided by 365 or 0.12 years

### RR estimates obtained using the HBsAg yield approach

The RR of HBV infection was estimated by applying a refined infectious WP estimate of 0.16 years (or 59 days) to the derived incidence estimates. Table 6 shows the incidence and RR estimates of HBV infection among blood donors at the Anhui blood center, which were obtained using the HBsAg yield approach. The estimated RRs for repeat donors, first-time donors, and all donors were 1 in 7804 (95% CI = 1:15800-1:5182), 1 in 1144 (95% CI = 1:1386-1:974), and 1 in 1853 (95% CI = 1:2219-1:1590), respectively (Table 6).

**Table 6:** The incidence and residual risk of TTHBV infection estimates in blood donations to Anhui from 2009 to 2011.

**Approach**	**Donor type**	**Incidence (/10^5^py**)**(1**)		**Window period**		Risk per 100,000 Donations (4=1*3)	**Rate of infectious donations**		
				**In days(2**)	**In years**		**Estimate**	**95%CI**	
					**(3=2/365**)		(1/4*10^5^)	**Lower limit**	**Upper limit**
**HBsAg Yield**	Repeat		79.3	59	0.16	12.8	1:7804	1:15800	1:5182
	First-time		540.6	59	0.16	87.4	1:1144	1:1386	1:974
	Total		333.9	59	0.16	54.0	1:1853	1: 2219	1:1590

## Discussion

China is currently facing seasonal blood shortages because the supply has not kept pace with clinical demand [19]. Recently, the system has changed from paid and employer-organized donations to voluntary donations; however, the emerging HIV epidemic, the growing syphilis epidemic, and the high prevalence of HBV and HCV among the general population pose a significant problem for Chinese blood banks. To maintain a safe supply of blood, it is important to gather epidemiological information regarding the prevalence and incidence of viral infection, the RR of TTIs, and TTI-associated demographic characteristics from both high-risk and low-risk individuals that volunteer as blood donors. The demographic characteristics of volunteers who donate blood at Anhui blood center are quite different from those of donors in Western countries [20]. For example, about 60% of donors are male, 62.8% are aged less than 25 years, most are single, and 69.2% are first-time donors (Table 2). As shown in Tables 3 and 4, repeat donors and single donors showed significantly lower rates of HBsAg-positivity than first-time donors, and married donors, respectively. To ensure an adequate blood supply that is free from HBV infection, it is critical to recruit suitable blood donors. In China, 70% of first-time donors return to give regular donations: this is critical to prevent further blood shortages and to increase blood safety.

Over the last two decades, the risk of TTHBV has steadily decreased due to the adoption of increasingly sensitive HBsAg tests, the implementation of anti-HBc screening (by some countries), improvements in the selection of volunteer donors, and NAT screening (performed either in mini-pools or, more efficiently, in individual samples). However, the risk of TTHBV in some countries remains higher than that of HCV and HIV-1, even after NAT became routine practice [4,5]. TTHBV still occurs even after the transfusion of blood that has tested negative for HBsAg. This is because blood may be donated by individuals during the WP of HBV infection, or by individuals that harbor chronic infection but have undetectable levels of HBsAg [6,7]. Of the currently available screening technologies, NAT shows the best potential for reducing the WP of infection and for identifying OBI. In some countries with a low prevalence of HBV, such as the USA, Germany, and Austria, donor blood is tested for HBsAg and anti-HBc, and for HBV genomic DNA using NAT. However, in China, which has a high prevalence of HBV, blood is screened for HBsAg using two different reagents that are provided in imported and domestically produced testing kits. In addition to the WP, there are other situations in which HBsAg may be undetected. For example, an individual may harbor OBI, or be infected with HBV harboring escape mutations associated with anti-HBs antibodies, or with HBV showing various deletions in the S region. Also, immune complexes formed by anti-HBs antibodies may mask the surface antigen and prevent detection. However, screening for anti-HBc antibodies, which may exclude most OBIs, is impractical in China, where the prevalence of anti-HBc antibodies is very high; this is because such screening may exclude many otherwise acceptable donors.

It is difficult to estimate the incidence of HBV infection and the RR with any degree of accuracy because of the transient nature of HBsAg expression, the suboptimal specificity of anti-HBc tests, false-positive HBsAg neutralization assay results, and the lack of NAT results for many units of donated blood. Because there is no routine confirmatory testing or anti-HBc testing in China, it is impossible to obtain epidemiological data regarding the prevalence, incidence, and RR for HBV. One study examined the RR of TTVI in Shenzhen, China, using a previously published method [21,22]. The estimated RR for HBV was estimated to be 1 in 17,501, with a lower RR for first-time donations than for repeat donations. This result was unexpected and contrasts with those obtained for Western blood donors. However, the authors assumed there is a constant likelihood of a currently infected donor being in the WP of the infection throughout the life time risk, which is not true. As indicated by their data, and the data obtained in the present study, the majority of donors are aged between 18 and 25 years. Thus, their calculated RR for HBV is likely to be underestimated. Using the HBsAg yield method, the current study estimated the incidence of HBV infection to be 79.3 per 100,000 py for repeat donors, 540.6 per 100,000 py for first-time donors and 333.9 per 100,000 py for all donors. The RR for HBV was estimated to be 1 in 7,804, 1 in 1,144, and 1 in 1,853 (Table 6) for repeat donors, first-time donors and all donors, respectively. While in western counties, the RR for HBV was estimated to be less than 1 in 200,000 with and without NAT [2,15,23]. The overall incidence and RR of HBV infection among Chinese blood donors is much higher than those among Western donors. NAT is the only testing method that can reduce the risk of blood-borne HBV transmission in high-endemic countries such as China. However, China is an economically unbalanced and developing country. There are more than 452 blood centers/banks that operate at three different levels: provincial (32), regional (321), and county (99). Most do not have access to and cannot afford NAT. Therefore, research into alternative methods and/or a reduction in the cost of NAT must be a priority. Fortunately, in 2011, NAT testing for HBV, HCV, and HIV was piloted in 11 selected blood centers, and will be implemented by all provincial blood centers over the coming years [19].

## Conclusion

The incidence and RR of HBV are much higher in China than in Western countries. The estimated RR for blood donations at Anhui could be substantially reduced by the implementation of improved HBsAg assays and by improving the cost-effectiveness of NAT.
